# Methodology of determining student’s cognitive styles and its application for teaching physics

**DOI:** 10.1186/2193-1801-3-449

**Published:** 2014-08-20

**Authors:** Igor V Grebenev, Ludmila B Lozovskaya, Ekaterina O Morozova

**Affiliations:** Physics Department, Nizhny Novgorod State University, Gagarin Avenue, 23, building 3, Nizhny Novgorod, Gagarin, Russia; Pedagogic. Physics Department, Nizhny Novgorod State University, Avenue, 23, building 3, Nizhny Novgorod, Gagarin, Russia; Physics Department, Indiana University, Bloomington, 727 E Third St., Swain Hall West, Indiana, USA

**Keywords:** Differentiation of learning, Cognitive styles, Testing

## Abstract

A psychological foundation of differentiation of learning on the basis of students’ individual characteristics (cognitive styles) with orientation to physics education in high school is discussed. The computer testing technique for preliminary determination of students’ cognitive styles is proposed. It is based on the assumed connection between test parameters and parameters of degree of manifestation of cognitive style. The combined method of refining students’ cognitive styles, based on the observations of their learning activity, is proposed. Personality traits, characterizing particular cognitive styles, are determined.

## Introduction

Modern concepts of the school and university education in Russia consider a problem of person-oriented education development as one of the most important problems. Development of the person-oriented education includes an implementation of individualization and differentiation of learning, and a formation of individual educational tracks. Due to propagation of exploratory and project-based learning, which is greatly influenced by cognitive characteristics of students, it is necessary to determine student’s cognitive potential. Preliminary investigations have shown that it is possible to select an individual plan of a learning process for every student, ensuring successful learning of a whole subject field, on the assumption that the optimal cognitive strategy can be determined and realized. Several bases of differentiation and methods of its implementations are known: biases, students’ abilities, learning curves. Differentiation by determining psychological characteristics of the students is an important direction, which allows combination of psychology, didactics and methodology of learning. This kind of differentiation can be based on identification of cognitive styles of the individual students. Cognitive styles are unique methods of processing information from the environment in the form of individual differences in perception, analysis, structuralization, categorization, and ongoing evaluation of the information. These individual differences constitute some typical forms of a cognitive appraisal, relative to which groups of people are similar and different from the other groups (Kholodnaya 2004; Hansen 1995; Sadler-Smith & Riding 1999). Cognitive style is an effective basis for conducting differentiated learning and developing corresponding methodologies, since it defines sustainable individual techniques of information processing, which are based on the degree of differentiation of perception.

### Problem statement

Experts in the field of psychology and pedagogy distinguish a sufficiently large number (about two dozen) of different cognitive styles. Among them, the most essential ones for the organization of differentiated learning process in STEM fields were defined in the previous works (McKenna 1984; Riding & Cheema 1991).

#### Field dependence/field independence

This cognitive style characterizes the ability of the subject to tune out the periphery of the field of perception. The field dependent people are guided by the external visible field of perception, and experience difficulties overcoming its influence. They need a lot of time to notice a necessary detail in a complex image. Field independent people, in contrast, tend to control the influence of visual impressions by relying on some internal criteria. They can easily overcome the influence of a visible field, and quickly find a detail in a complex image. In a more general formulation, this style characterizes subject’s orientation of perception and thinking either to the external factors (tendency to be field dependent) or internal factors (tendency to be field independent).

#### Impulsivity/reflectivity

Impulsivity/reflexivity characterizes individual differences in the tendency to make decisions quickly or slowly. Most evidently it can be seen in the multiple-choice situations. Impulsive people tend to react quickly in such situations, they put forward the hypotheses without analyzing all possible alternatives. In contrast, for reflexive people slow pace of response is typical. They test the hypotheses and more accurately specify the information. Their decisions are based on a thorough preliminary analysis of the signs of alternative objects.

It is generally accepted that these classifications are orthogonal to each other in the sense that, for example, field dependent students with equal success can possess an impulsive as well as a reflective type of response. Therefore, generally, four groups of students could be distinguished: field dependent - impulsive (FD-I), field dependent - reflective (FD-R), field independent - impulsive (FI-I), field independent - reflective (FI-R).

Even though, cognitive styles and their capacity to determine a learning process efficiency of each individual student have been described a long time ago, positive experience of the real differentiation of learning based on cognitive styles was quite little in Russian schools. It can be explained by several reasons:

-  a complexity of the testing procedures and ambiguous interpretation of their results;
-  abstract basis of the tests, not quite related to the upcoming learning activities of the students;
-  identification of the students’ psychological characteristics based only on the test results, insufficient accounting for the learning activities of the students;
-  predominance of the general recommendations on the organization of the teaching, excluding concrete learning activities in each particular subject;
-  capture of the only general characteristics of cognitive style, without internal specification of the psychological characteristics that underlie learning activities.

Method of determining main cognitive styles of the students is based on the operations with abstract objects that are not related to the actual teaching activities (McKenna 1984; Witkin et al. 1977). Therefore, the ascertained psychological type might not be displayed in the learning process of a particular subject in the expected form. A computer test should be supplemented by the observations of the students’ learning activities, which can give the weighty arguments for determining a particular cognitive style of a student and for a further variant of the educational process.

Previous studies have identified the psychological characteristics of the students’ activity, specific to the certain combinations of cognitive styles and which are the most pronounced in learning physics (Table 1). The table is divided into four sections which indicate the prevalence of the corresponding type of differentiation field (FD/FI) and the type of response (I/R), so that each quadrant represents one of the four cognitive types: (FD-I), (FD-R), (FI-I), (FI-R). Each quadrant contains a detailed description of the learning activities of the students with particular cognitive styles, identified in our experiment.

**Table 1:** **Psychological characteristics of students’ learning**

I	
Student answers are poorly thought-out and hasty.	Student answers are poorly thought-out and hasty.
Student is inclined to ignore the important but less visible details, while working with the textbook.	Student is able to focus on the important details, while working with the textbook.
While retelling teaching material, student often misses the main point, adds unnecessary information and might get confused by it.	While retelling teaching material, student clearly highlights the main points.
When a teacher explains teaching material, student tends to anticipate and voice teacher’s thoughts.	When a teacher explains teaching material, student tends to anticipate and voice teacher’s thoughts.
Student solves simple problems quickly, difficult problems he solves rather quickly as well, however, solutions are often incorrect.	Student solves simple problems quickly, difficult problems he solves rather quickly as well, however, solutions are often incorrect.
Student reads the conditions of the problem inattentively.	Student reads the conditions of the problem inattentively.
When student receives information, he often asks to repeat the information and requests additional information.	Student seeks to manage when he is performing laboratory work in a group.
Student can not gain knowledge out of the demonstration by himself.	Student is able to draw conclusions out of the demonstration.
Student often hesitates, in a group work he is guided by the opinion of the others.	Student is not inclined to take someone else’s point of view when he is working in a group.
**FD**	**FI**
Student prepares his answers carefully and for a long time, he acts carefully.	Student prepares his answers carefully, he acts carefully as well.
Student is inclined to ignore the important but less visible details, while working with the textbook.	Student focuses on the important details, while working with the textbook.
While retelling teaching material, student often misses the main point, adds unnecessary information and might get confused by it	While retelling teaching material, student often clearly highlights the main points, his answers are connected and logical.
When a teacher explains teaching material student tries to understand teacher’s train of thought and take notes.	When a teacher explains teaching material, student tries to understand teacher’s train of thought, but only take notes on the important information or writes down nothing.
Student solves simple tasks quickly, difficult tasks he solves slowly.	Student solves simple tasks quickly, difficult tasks he solves slowly.
Student tries to read the conditions of the problem carefully, however, even this does not help him to found the key moments for solving the problem.	Student reads the conditions of the problem carefully enough, he finds the key to problem solving without much difficulty.
When student has a test he does not have time to complete it.	Student tends to solve problems on his/her own, he/she successfully solves problems by analogy.
Student can not gain knowledge out of the demonstrations or experiments by himself.	Student tends to take the initiative when he does the laboratory works.
Student demonstrates a lack of confidence, while working in a group, he is inclined to let other students to make decisions (i.e. he follows more confident students).	Student is able to draw conclusions out of the demonstrations and experiments.
	Student demonstrates confidence, resoluteness and independence.
**R**	

Given signs of the degree of manifestation of certain psychological characteristics could be useful for the qualitative determination of cognitive styles of the individual students. They indicate possible features of the optimal cognitive activity for the students. However, observations are not enough for the accurate determination of the individual psychological characteristic and organization of the comfortable learning process in a concrete subject. They should be combined with the computer testing methods adapted to the important features of the students’ cognitive activity in a concrete subject.

A primary goal of our work was a creation of a new combined approach for determining cognitive characteristics of the students, which would more reliably predict the influence of these characteristics on the learning activity.

### Method of psychological testing

Traditional tests (Witkin et al. 1977; Kagan 1966; Cools and Van den Broeck 2007) created for measuring the selected parameters of cognitive style are as follows:

-  "field dependence – field independence" is diagnosed by means of Witkin’s embedded figures test. A test subject is required to find some simple figures placed on the more or less complex background;
-  "impulsivity – reflectivity" is measured by the method of J. Kagan. According to this method a test subject compares the contour image of the object with several similar images and finds the identical to the pattern image. Response time is measured and the number of the wrong decisions is counted during the test. A subject, who completed the task with a few mistakes, but spent a lot of time thinking about the answers, belongs to the category of reflexive subjects. The opposite combination of signs is an indicator of impulsivity.

**Figure 1:**
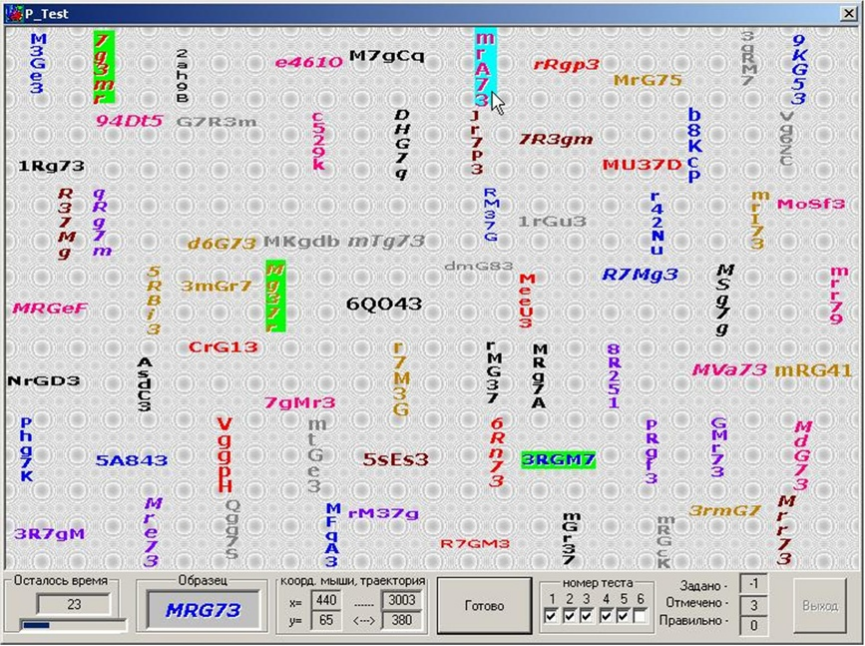
**User screen of the computer-based test for the identification of cognitive styles.**

It was necessary to create a new version of the test oriented to the use of its results for teaching physics due to the following reasons:

-  Factor structure, revealed during the processing of the test data, which provides the basis for the identification of cognitive styles, is not invariant with respect to the type of the test tasks. This statement dictates two requirements, which restrict the test tasks from the opposite sides. First, the materials of the test, which results are planed to be used for designing the educational process in a concrete subject, should not rely on the level of students’ achievement in this subject and generally on their training level. Second, the nature of the tasks and students’ activities during the test should reveal personal qualities, cognitive features and capabilities that are the most important and the most significant in the learning process in a concrete subject.
-  The designed test is orientated to the specific types of cognitive activity for learning physics. The greatest importance is attached to the operations of perception and processing symbolic and numeric information in the abstract representation. Operations with the visual images that form the basis for the typical tests using pictures, although are significant for teaching physics, but less important than described above tasks of processing symbolic form of data representation. It is extremely important to test not only subjects’ ability to percept and store information, but to a greater extent their ability to transform and consciously process this information.
-  Not only and not so much the test result should be studied, but the operating component of the students’ activity during the test execution. For the investigation of the persons’ individual activity, the test is made in such a way that the basic parameters of the activity itself, peculiarities of the goal achievement process by each individual would be available for the analysis and would have a numeric expression.
-  Test results should enable the use of the factor-correlation analysis for the numerical evaluation of the degree of manifestation of cognitive style parameters (Grebenev et al. 2005).
  Test is a computer program in a dialog box (Figure 1) consisting of six tasks which complexity increases with the number of the task. It is necessary for the subsequent analysis of the dynamics of the change of the measures in the process of the test execution. Rows of the equal length consisting of various characters are arranged on the information field with the background. A pattern string is located at the bottom of the dialog box. The length of the strings, their quantity and the location variability increases with the number of the test task. The goal is to select all strings, which consist of the same letters and numbers as the pattern string, including all variants of the permutations of characters, variations of color, size and line orientation.

Besides the number of hits, a set of the following parameters is recorded during the test:

- execution time;
- average time spent finding the set of characters;
- length of the mouse trajectory during the task execution;
- average length of the mouse displacement vector during rows selection;
- variance of search time and coordinates.

Test materials (the results and the process of the tasks execution) are raw data and are processed by means of the factor-correlation analysis, in our case using SPSS software package. Application of the factor-correlation analysis for data processing allows obtaining a structure of personality traits, based on the observable variables, and gives a numerical estimate of the degree of manifestation of each trait. Parameters such as the length of the mouse trajectory during the test execution, the average length of the mouse displacement vector, along with the number of errors will indicate the relative rationality of the activity strategy of a test subject due to a bigger or smaller active field of perception. Thereby, it is assumed that the relation of these variables with the parameter of the field dependence exists –the shorter trajectory with the same result indicates the greater degree of manifestation of field independence of the individual. Parameters of time and coordinate variance of the correct stimulus search should indicate the relative rationality of the activity strategy of the individual and its measure of impulsivity.

Reflectivity of the cognitive strategy is revealed by the fact that as the complexity of the task increases, the time spent on information processing (search time of the right stimulus, time till pressing "Finish" button, («Готово»)) increases as well. Variance of search time and coordinate for reflective students will be larger.

Accordingly, such test parameters as the average search time of the correct stimulus, variance of search time, the time till finding the first correct character set, the time till pressing the "Finish" button («Готово»), and the full execution time would be responsible for the response type (I/R). Test parameters such as the length of the mouse trajectory during the test execution, the average path length between mouse clicks, the number of misses would be responsible for psychological differentiation (FD/FI).

Factors were identified using SPSS package by means of the principal components method "Principal components" with subsequent varimax rotation "Varimax normalized". The number of factors was identified by calculating the smallest eigenvalue (λ ≥ 1). The data containing the analysis of the test results obtained from students of 9–11 grades of Nizhny Novgorod schools (270 persons) is stated below. Matrix of factor loadings after varimax rotation is as follows (Table 2).

**Table 2:** **Matrix of factor loadings**

Variable (sign)	Factor 1	Factor 2
Mouse trajectory	**0.876**	0.071
Average length of the displacement vector	**0.717**	0.097
Time of the beginning of test task	-0.371	**0.885**
Execution time	-0.558	-0.401
Variance of search time	0.459	**0.733**
Variance of coordinates	**0.881**	0.216

Significant values of factor loadings are shown in boldface.

Stated result of the standard procedures of factor-orrelation analysis suggests that the test variables determine two factors. The first factor is formed by the variables, such as the mouse trajectory, the average length of the mouse displacement vector and variance of coordinates. The second factor is formed by the variables, such as the time of the beginning of the test task and variance of search time during the task execution.

The first factor explains a part of the total variance equal to 2.71 (Eigenval) or 45.2%. Both factors explain 4.07 (Cumul. Eigenval) of variance, or 67.85%. This variance is enough to describe the variation of the signs.

The first factor is related to the coordinate characteristics, and it can be concluded that this factor is associated with field dependence - field independence. Large value of such variables as the mouse trajectory, variance of coordinates and the average length of the displacement vector suggests field dependence of a test subject, since he/she is lost in a large information field, and can not identify the necessary part.

The second factor is related to the timing characteristics, this suggests that it is connected to the type of response. Small value of this factor is associated with impulsivity. The individual spends little time thinking over the information and almost immediately tries to solve the problem.

For the further discussion, it is important to note that there are negative relations of the factors (interpreted as field independence) with the time characteristics, and negative loads of execution time (time till clicking "Finish" button) on both factors. This data demanded further, more thorough analysis of the internal structure of such characteristic as field independence.

Since this study is ultimately aimed to improve the effectiveness of teaching physics, it is important to identify students’ cognitive style in a particular academic work. Therefore, a combined method, which is a sequence of actions of a psychologist and an educator, was developed. This method consists of the following steps:

- conducting the computer test and analyzing its results;
- monitoring the learning activities of the students according to the Table 1;
- analysis of the written work;
- analysis of the oral answers;
- pedagogical council.

Thus, the computer test allows determining the formal parameters of students’ cognitive styles. On the basis of these parameters they will be pre-assigned the corresponding psychological characteristics, which will be then confirmed or adjusted by further stages of the methodology, based on the observations and analysis of their educational work. Next, pedagogical council allows considering the opinion of physics, mathematics and natural science teachers and school psychologist, and assigning cognitive style to an individual student. In case of the divergence of opinion, pedagogical council may suggest to pay more attention to this student before assigning him/her appropriate cognitive style.

Correlation value between test and empirical data (the results of observations and of pedagogical council) are not lower than 0.65-0.7 both for field dependence parameter and impulsivity - reflexivity parameter. This, besides other things, proves a validity of the proposed variant of the computer test.

At the same time, obvious "mistakes" in the identification of cognitive style of the individual students according to the test were noted while comparing the test results with the results of observations and pedagogical council. This could not be explained by the errors of mathematical processing. Test performance of some students is close to the median. Integral estimate of cognitive style by itself may be insufficiently accurate to build an effective educational track for a particular student in a real educational process. All this also suggests a necessity of establishing a more detailed psychological structure of the type that is related, for example, to field independence, according to the test results.

### Detailing features of cognitive activity of students with different cognitive styles

Further investigation is aimed to a more detailed identification of the internal structure of the most important for learning cognitive characteristic – field independence. Under the same identified cognitive characteristic of the student, his/her psychological profile can vary significantly. The same observed behavior, interpreted as a sign of a certain cognitive style in learning activity, can be a consequence of the various complexes of inner characteristics. Therefore, for the formulation of more precise recommendations on the organization of differentiated variants of the educational process it is necessary to find the important elements of the internal structure of these integral estimates, conditions under which, for example, field independence could be revealed.

A correlation of field independence/field dependence parameter with the features of cognitive activity, serving as a condition of the presence of one or another cognitive style, was investigated on the basis of Yu.Borisova works (Borisova Yu and Grebenev 2003).

1. Since cognitive style, differentiation of the field, is characterized by the ability to quickly or slowly tune out the periphery of the field of perception, it includes an important component of productivity of the brain activity: response time. Response time is defined by the flow rate of intellectual operations, which is determined in our study through the timing characteristic of the execution of the embedded figures test (Oltman et al. 1971). It should be noted that we do not interpret response time, identified by this test as a thinking rate, since the reaction of the person could occur at unfinished thought processes.
2. Ability to highlight the main idea in the text is the essential condition of the presence of particular cognitive style. A skill to highlight key information is a complex process of thinking, which is based on the capacity for analysis, synthesis and abstraction. It influences the identification of characteristics of a particular cognitive style. This ability is also important in the learning process. For example, a student, who is capable to highlight the main idea in obtained information, will show the best results in the test on field dependence and will be able to learn the content better. He will not get confused in the flow of obtained information, and will memorize and learn only the main moments. Unlike him, a student, who is unable to select the main ideas, will be suppressed by the information flow. He will need to remember all the received information, which is impossible. Moreover, the student hardly can paraphrase what he heard, therefore, he needs a personal assistance of teachers or classmates in a group work.
  Considered indicator is determined by means of achievement test, directed to understanding the information and highlighting the main idea. This test is made on the basis of the physics teaching materials according to the method described by (Anastasi & Urbina 1997).
3. Another important condition for identifying particular cognitive style is independence of thinking of test subjects. Independence of thinking indicates the ability to efficiently use social experience, it is also clearly correlated with field independence. Independence of thinking was determined in our research on the basis of monitoring the process of the execution of the learning tasks by the students, and also on the basis of how often and why they ask a teacher or classmates for help.

Analysis of data from the experiment (270 test subjects, students of 9–11 grades) allows selecting 5 types of learners.

- Type 1 (39 students) is characterized by field dependence, short response time, reliance on outside help, inability to highlight key information. These students typically finish the embedded figures test quite fast, but make a lot of mistakes by showing the wrong simple shapes.
- Type 2 (78 students) is characterized by field dependence, long response time, reliance on outside help, inability to highlight key information. These students made a lot of mistakes in embedded figures test even working on the tasks for a long time. Students often were unable to finish the task, because they could not see a simple figure in a complex background.
- Type 3 (44 students) is characterized by field dependence, long response time, reliance on outside help, ability to highlight key information. These students did the embedded figures test tasks quite slowly, but in most of the cases correctly.
- Type 4 (66 students) is characterized by field independence, short response time, self-consistency, ability to highlight key information. These students find simple shapes in a complex image quickly and accurately and in a relatively short time.
- Type 5 (43 students) is characterized by field independence, long response time, self-consistency, inability to highlight key information. These students are able to find a simple shape in a complex image, but they need quite a lot of time.

Correlation between the index of field dependence/field independence and the ability to highlight key information (correlation coefficient rs = 0.77, significance level p <0.05) and self-sufficiency (rs = 0.66, significance level p <0.05) was found.

As a whole, our findings suggest the following:

-  independence of mind is a basic condition of field independence of the students;
-  inability to highlight key information and reliance on outside help are the basic conditions for field dependence of the students.

The most interesting negative correlation in this experiment was found between field independence and response time (rs = - 0.71, significance level p <0.05).This correlation has already been indicated in Table 2.

Thus, all three conditions given to describe the features of cognitive style, field differentiation, turned out to be connected:

- the more person is field dependent the less he is able to highlight key information, less independent in information search, and analysis and has longer response time.
- the more person is field independent the more he is self-sufficient, more capable to select main ideas, and has shorter response time.

Negative correlation of field independence with the response time was revealed again on the next step of the research as a link with the successful learning (Table 3). This reflects the influence of another dimension - the type of response (impulsivity/reflectivity). Short reaction time reflects the measure of impulsivity evidence. This result proves the necessity to consider a combination of cognitive styles and confirms the greatest learning efficiency of FI-R students. At the same time, this can be seen as an argument against the hypothesis of orthogonality of considered cognitive styles.

**Table 3:** **Correlation coefficients of indicators of cognitive style and academic performance of students**

Indicators of cognitive style	Academic average for different subjects				
	Physics	Chemistry	Mathematics	Russian language	History
Index of field dependence/field independence	0,55	0,55	0,49	0,25	0,44
Response time	-0,43	-0,40	-0,37	-0,22	-0,43
Ability to highlight key information	0,72	0,63	0,63	0,46	0,59
Self-sufficiency	0,72	0,66	0,64	0,28	0,63

Correlation of indicators of cognitive style with mean value of academic performance of the students is well known (Hansen 1995; Billington et al. 2007) (the Russian data (Lozovskaya 2011; Malykh et al. 2012)). We identified the influence of various indicators of cognitive style on success in learning natural sciences and arts. Table 3 contains correlation coefficients between various indicators of cognitive style and academic performance in various subjects.

Students characterized by field independence, self-sufficiency, ability to highlight important information and reasonably quick reaction capability (type 4), have the highest indices of academic performance in natural-scientific subjects. Students of the opposite type, who are characterized by field dependence, reliance on outside help, inability to highlight important information, long reaction time (type 2), have the lowest indices.

## Results and discussion

Physical and mathematical education traditionally raises specific and strict demands for cognitive sphere of students. These demands are determined by a combination of inductive and deductive stages of the educational process, a study of logically complete copies of the physical theories, and a large share of independent practical exercises. Influence of field independence on arts is not as great as on sciences. The causes of positive correlation between learning efficiency and the degree of manifestation of FI/R combination of cognitive styles have been revealed in the results of the study, described above. Field independence helps the students to see not only studied concepts and laws, but their conceptual environment, existent relations by virtue of the more analytical and differentiated approach to information processing. As a whole, field independence allows students of this cognitive style to see a wide adjoining conceptual field, and reflexivity allows them to follow even implicit logic of disclosure of conceptual links in the laws. For field dependent students, conceptual environment, existent relations and genesis are not apparent, and can not be learned in the absence of teacher’s special work. This makes these groups of students less capable of applying concepts and laws in a situation different from that in which they were formed. Therefore, FI-R pole of cognitive style characterizes the ability of the students to independently generalize and organize data, identify cause-and-effect relationships, trace the genesis of the concepts and laws, and effectively apply their knowledge in the future. The ability of the students to transfer their knowledge to a new cognitive situation is a sign of a full understanding of learning contents of the disciplines, in which the deductive element of the learning content predominates.
